# Body size dictates physiological and behavioural responses to hypoxia and elevated water temperatures in Murray cod (*Maccullochella peelii*)

**DOI:** 10.1093/conphys/coac087

**Published:** 2023-01-28

**Authors:** Darren McPhee, Jabin R Watson, Doug J Harding, Andrea Prior, James H Fawcett, Craig E Franklin, Rebecca L Cramp

**Affiliations:** School of Biological Sciences, The University of Queensland, Brisbane, Queensland, 4072, Australia; School of Biological Sciences, The University of Queensland, Brisbane, Queensland, 4072, Australia; Queensland Department of Regional Development, Manufacturing and Water, 203 Tor St., Toowoomba, Queensland, 4350, Australia; Queensland Department of Regional Development, Manufacturing and Water, 203 Tor St., Toowoomba, Queensland, 4350, Australia; Queensland Department of Regional Development, Manufacturing and Water, 203 Tor St., Toowoomba, Queensland, 4350, Australia; School of Biological Sciences, The University of Queensland, Brisbane, Queensland, 4072, Australia; School of Biological Sciences, The University of Queensland, Brisbane, Queensland, 4072, Australia

**Keywords:** thermal plasticity, thermal acclimation, PCT_max_, Murray-Darling Basin, maximum thermal tolerance, hypoxia threshold, gill ventilation rate, fish death events, CT_max_

## Abstract

Increasing drought frequency and duration pose a significant threat to fish species in dryland river systems. As ectotherms, fish thermal and hypoxia tolerances directly determine the capacity of species to persist in these environments during low flow periods when water temperatures are high and waterbodies become highly stratified. Chronic thermal stress can compound the impacts of acute hypoxic events on fish resulting in significant fish mortality; however, it is not known if all size classes are equally susceptible, or if the allometric scaling of physiological processes means some size classes are disproportionately affected. We investigated the physiological responses of Murray cod (*Maccullochella peelii*) over a four-fold body size range (0.2–3000 g) to acute changes in water temperature and oxygen concentration following 4 weeks of acclimation to representative spring (20°C) and summer (28°C) water temperatures. We recorded maximum thermal tolerance (*CT*_max_), oxygen limited thermal tolerance (P*CT_max_*), lowest tolerable oxygen level (as the oxygen level at which lose equilibrium; O_2,LOE_)*,* gill ventilation rates and aerial surface respiration threshold, blood oxygen transport capacity and lactate accumulation. Acclimation to elevated water temperatures improved thermal and hypoxia tolerance metrics across all size classes. However, body size significantly affected thermal and hypoxia responses. Small *M. peelii* were significantly less hypoxia tolerant than larger individuals, while larger fish were significantly less thermal tolerant than smaller fish. Hypoxia constrained thermal tolerance in *M. peelii*, with both small and large fish disproportionally compromised relative to mid-sized fish. Our findings indicate that both very small/young (larvae, fry, fingerlings) and very large/older *M. peelii* in dryland rivers are at significant risk from the combined impacts of a warming and drying climate and water extraction. These data will inform policy decisions that serve to balance competing demands on precious freshwater resources.

## Teaser

Increased temperature and hypoxia are threatening dryland river waterholes that serve as critical refugia for native fish. Australia’s largest freshwater fish Murray cod (Maccullochella peelii) showed body-size-dependent differences in thermal and hypoxia tolerances, indicating that small fish inhabiting waterholes are at increased risk from climate-associated changes in filling and drying regimes.

## Introduction

Changes to climatic conditions in recent decades threaten to reduce the functionality of freshwater ecosystems and the biodiversity that they support (Bond *et al.,* 2008; Pittock and Finlayson, 2011). Unprecedented rates of global warming have contributed to an increase in the frequency, intensity and duration of droughts, as well as increased rates of evapotranspiration, and significant increases in mean water temperatures (Aldous *et al.,* 2011). Congruently, direct human impacts on these systems are increasing as demand rises for water use in agricultural, domestic and industrial sectors. This ongoing competition between maintaining water security and ecological health has placed intense pressure on species that depend on freshwater ecosystems, particularly those ecosystems with naturally dynamic flow patterns. In Australia, the Queensland Murray-Darling Basin is such a system, experiencing periods of high flow followed by protracted episodes of low flow, no flow and droughts. During periods of no flow, connectivity throughout the river system diminishes, with the watercourse forming a mosaic of spatially isolated waterholes that serve as critical refugia for native freshwater fish species (Balcombe *et al.,* 2006; McNeil *et al.,* 2013). Protracted periods of no flow associated with a rapidly changing climate and other anthropogenic pressures place waterholes under increased pressure as refugia for aquatic species (Bond *et al.,* 2015). Water quality in refugial pools is often very poor, ranging from severely hypoxic to anoxic in some places (Negus *et al.,* 2019). Waterholes can become highly oxygen-stratified during periods of no flow, with oxygen depleted sub-surface layers and a much smaller oxygenated surface layer. These highly stratified waterbodies are particularly susceptible to acute destratification during flow events or storms, which can result in the hypoxic/anoxic subsurface layers being rapidly redistributed throughout the water column, killing hundreds of thousands of fish in a matter of hours (Wong *et al.,* 2018, Moritz *et al.,* 2019). Understanding and managing the threat of increasing drought frequency and severity on freshwater fish species requires a greater understanding of the capacity of different species to resist or tolerate changes in waterhole water quality, particularly high temperatures and reduced oxygen availability.

Environmental temperature and oxygen partial pressure are the key factors underpinning the behaviour and physiology of fish (Reynolds and Casterlin, 1980; Lannig *et al.,* 2004; McArley *et al.,* 2020). As ectotherms, fish performance and oxygen demand increases with increasing temperature up to a maximum point, after which performance declines. Likewise, aquatic oxygen availability influences the capacity of fish to sustain performance aerobically. In the short term, fish exposed to increased water temperatures and/or hypoxia can adjust behavioural and physiological processes to compensate for the effects of reduced oxygen availability or increased oxygen demand. Behavioural adjustments can occur quickly, for example, by moving to habitat with more favourable conditions (McNeil and Closs, 2007) and/or performing aquatic surface respiration (ASR), whereby fish gulp at the thin layer of well-oxygenated water at the air-water interface (Small *et al.,* 2014; Dixon *et al.,* 2017). Physiological responses, such as reducing the thermal sensitivity of a trait, increasing oxygen uptake capacity or depressing metabolic demand, may take longer to develop (Crawshaw, 1976; Farrell and Richards, 2009; Ern *et al.,* 2014; Glazier, 2014). Sustained exposure to elevated temperatures and hypoxia can result in the acclimation/acclimatization of traits whereby physiological processes are shifted to optimize performance under the prevailing environmental conditions (Anttila *et al.,* 2015; Zhou *et al.,* 2019). In some cases, physiological changes that occur in response to high temperature can affect the response of the animal to other stressors (e.g. Burleson and Silva, 2011, McBryan *et al.,* 2016; Nilsson *et al.,* 2010, McArley *et al.,* 2020; Gomez Isaza *et al.,* 2020). For example, in some fish, exposure to high temperatures can lead to increased gill surface area which improves their capacity to uptake oxygen at high temperature, but also improves their ability to tolerate low oxygen environments (Sollid *et al.,* 2005; Sollid and Nilsson, 2006; McBryan *et al.,* 2016). As such, the management of waterhole water quality and the implications for fish habitability need to consider how chronic exposure to one stressor may influence the response of fish to other co-occurring stressors.

Changes in waterhole water quality during no flow periods can affect fish at all stages of development and concurrent changes in body size can influence how fish may respond to these stressors (White *et al.,* 2006). Inherent physiological differences between fish at different life stages can contribute to their ability to respond to changing environmental conditions. For example, larvae or juveniles that may be allocating energy towards growth and development may have a lower capacity to withstand change. Similarly, adults that are of reproductive age may be investing energy resources into egg production, also reducing their capacity to respond to a changing environment.

The effect of body size on fish hypoxia tolerance has been widely studied, and while body size dependent hypoxia tolerance is highly variable across fish species, for many (but not all), larger individuals tolerate hypoxia better than smaller ones (Nilsson and Ostlund-Nilsson, 2008). For example, in the freshwater oscar, *Astronotus ocellatus*, larger individuals had a longer survival time under hypoxic conditions relative to smaller fish and were able to regulate their aerobic metabolism (P_Crit_) at lower oxygen saturation levels (Sloman *et al.,* 2006). Thermal tolerance limits are also correlated with body mass in some, but not all, fish species. For those species that do show mass-dependent thermal tolerance, thermal tolerance generally declines with increasing body mass (e.g. Recsetar *et al.,* 2012; Underwood *et al.,* 2012; Messmer *et al.,* 2017; Bartlett *et al.,* 2022). Fish are the only group of vertebrates that can vary in body mass within an individual’s life history by more than eight orders of magnitude (Glazier, 2005). The relationship between most physiological traits and body size are not linear, and instead scales allometrically. If particular physiological traits that underpin a species’ ability to deal with an environmental stressor scales allometrically with body size, then some size classes may be more tolerant of changes to this stressor, while some may be disproportionately susceptible. To accurately assess the capacity of a species to tolerate exposure to environmental stressors it is important to consider how tolerances change with body size, especially with species that vary significantly in size across their life histories.

Rapid environmental change, coupled with reduced waterway connectivity and water abstraction is affecting a range of fish species in the Murray-Darling Basin, but perhaps Murray cod (*M. peelii*) more so because of their large size and slow growth rate. *Maccullochella peelii* are a long-lived, freshwater species, endemic to the Murray-Darling Basin (Leigh and Zampatti, 2013) and the largest freshwater fish in Australia, with some specimens recorded weighing >100 kg (Rowland, 1983). *Maccullochella peelii* have suffered significant population declines since European settlement because of overexploitation, habitat loss and river regulation (Humphries and Winemiller, 2009). Several populations in the Murray-Darling Basin have also experienced mass fish deaths in recent years because of exposure to severely hypoxic waters and increased water temperatures (Moritz *et al.,* 2019). Because the seasonal timing of the development of these conditions coincides with post-spawning juvenile recruitment in *M. peelii* (late spring and summer) (Rowland, 1983), it is highly likely that a significant range of life stages could be exposed to the extreme environmental changes that accompany protracted low flow periods. If larval or juvenile *M. peelii* have a reduced capacity to withstand or resist these stressors relative to larger individuals, it highlights a serious concern for this species as losses of these smaller size classes may go undetected until they begin to affect the species at a population level (Downes *et al.,* 2021). This study aimed to investigate the influence of body size on the physiological tolerances of *M. peelii* to high temperatures and hypoxia following acclimation to high water temperatures. To determine the relevant experimental levels, temperature and aquatic oxygen levels were measured at three waterholes during spring and summer in the Queensland Murray-Darling Basin. *M. peelii* (size range = 0.2–3000 g) were acclimated to either an average early spring temperature (20°C) or an average late summer temperature (28°C) and their maximum thermal tolerance (*CT*_max_) and oxygen limited thermal tolerance (P*CT_max_*) were measured. Hypoxia tolerance was assessed using loss of equilibrium (O_2,_LOE) tests, gill ventilation rates (GVRs) and the aquatic oxygen saturation level at which *M. peelii* performed aquatic surface respiration (ASR*_crit_*). We also measured oxygen transport metrics (haemoglobin and haematocrit) and blood lactate levels in response to an acute hypoxia event. These traits were selected for their known sensitivity to both high temperature and low aquatic oxygen saturation levels, and their capacity to change following a period of acclimation. It was hypothesized that chronic exposure to higher water temperatures would lead to improvements in both the thermal and hypoxia tolerances of *M. peelii*. Due to the allometric scaling of physiological responses, it was further hypothesized that larger animals would be more tolerant of high temperatures but less tolerant of low aquatic oxygen saturation levels than juvenile *M. peelii.*

**Figure 1:**
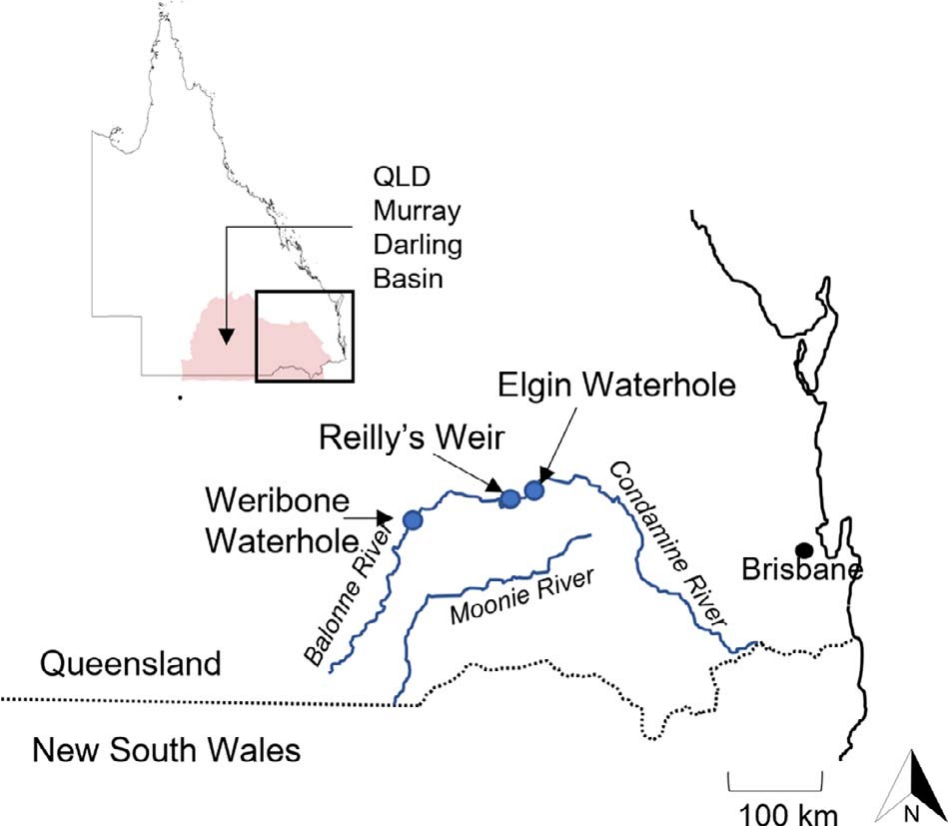
Location of field sites in the Condamine-Balonne River in the Queensland Murray Darling Basin.

## Materials and methods

### Waterhole quality assessments

**Figure 2:**
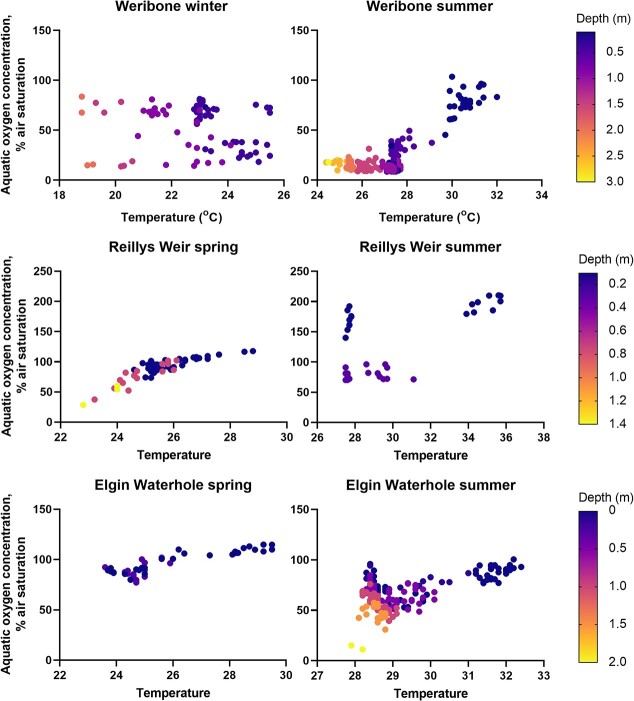
Relationship between water temperature (°C), aquatic oxygen level (as % air saturation) and water depth (m; symbol colour) in three waterholes along the Condamine-Balonne River in the Queensland Murray Darling Basin in spring (left panels) and summer (right panels) of 2019–2020. Each row of panels is representative of an individual waterhole. Data points at each depth represent values collected along a horizontal transect in both the early morning and at midday on one day in each season to capture the range of conditions occurring over that day.

To investigate the diel and seasonal patterns of water temperature and oxygenation that *M. peelii* can experience during the warmest parts of the year, three waterholes in the Condamine-Balonne River in the Queensland Murray Darling Basin were surveyed in both Spring and Summer of 2019–2020 over two separate 24 h monitoring periods (Fig. 1). The measurements recorded during these surveys were used to inform experimental exposure levels for *M. peelii.* The waterholes were selected on the basis that they represented varying levels of habitat quality and were known to support *M. peelii*. Presence of *M. peelii* in waterholes was confirmed by locating fish that had previously been acoustically tagged by the Department of Regional Development, Manufacturing and Water (DRDMW) using a mobile acoustic receiver (Vemco VR100, Nova Scotia, Canada). Aquatic oxygen saturation measurements (as % air saturation) were made using a temperature, salinity and pressure compensated YSI aquatic oxygen logger (YSI Professional Plus, Xylem Inc., United States), calibrated before each sampling period. Water temperature and aquatic oxygen levels were measured early morning and mid-afternoon along horizontal and vertical transects across all three waterholes. Horizontal transects were taken across the waterhole, in the centre of the waterhole and within 3 m of the banks on either side of waterholes. The frequency of horizontal transects varied between 10 and 50 m due to significant differences in waterhole length. At each sampling point, measurements were taken every 0.5 m down the vertical water profile until reaching the bottom. The geographical co-ordinates (latitude and longitude) were recorded for each corresponding sampling point using a GPS (Etrex 10, Garmin, Australasia Pty. Ltd, Australia). All observations are provided in Fig. 2.

### Fish collection and husbandry

Fertilized *M. peelii* eggs (*n* = 200) were sourced from Condabilla Fish Farm, Chinchilla, QLD. Eggs were immediately transferred to the University of Queensland, Australia, and placed into a 1000 l recirculating holding system, with mechanical and biological filtration and ultraviolet sterilization. This system comprised of twelve 40-l glass aquaria (60 x 25 x 25 cm). Eggs were evenly distributed into three tanks and maintained at 20°C with a fixed photoperiod of 12:12 h light to dark. Tanks containing eggs were treated with methylene blue (Aquasonic Ltd, Australia) to prevent fungal growth. Methylene blue treatment was halted following hatching, and once yolk sacks were absorbed, *M. peelii* larvae were fed to satiation twice daily with live brine shrimp nauplii (*Artemia spp*.). Brine shrimp were replaced by commercial bloodworms (Hikari, Kyorin Co. Ltd, Japan) once *M. peelii* were of adequate size. Larvae were reared for a minimum of eight weeks before testing.

Juveniles and sub-adults (*n* = 92, mass = 2.5–769 g) were purchased from multiple commercial hatcheries (9Dorf Farms, Lilydale and Granite Belt Fish Hatchery, Severnlea, QLD, Australia). Adult fish were sourced in 2015 from a commercial hatchery (Ausyfish, Childers, QLD, Australia) as juveniles and reared to adult size in our facility. Smaller fish (2.5–480 g) were evenly distributed between twelve 40-l holding tanks based on body mass. Sub-adult (*n* = 22, mass = 480–650 g) and adult fish (*n* = 8, mass = 1.49–2.55 kg) were housed in separate 5000 l recirculating tanks. Juvenile and adult fish were fed commercial pellets daily (EP6 and EP8, Otohime, Marubeni Nisshin Co., Japan). All holding tanks were maintained at 24 ± 1°C with a fixed photoperiod of 12:12 h light to dark. Tanks were cleaned weekly, with continuous water exchange supplied by carbon-filtered Brisbane tap water on a flow-through system exchanging 20%–50% of the system volume in 24 h. Prior to experimentation, all fish were fasted for 24 h to achieve a post-absorptive state. Experiments were conducted in compliance with the University of Queensland animal ethics requirements (permit no. 249/17).

### Tagging

Due to logistical limitations of housing high numbers of large individuals, some *M. peelii* were allowed to grow and were retested over time. To account for potential individual effects, all fish larger than 5 g were tagged with Visible Implant Elastomer tags (Northwest Marine Technology, USA). After a 24 h fasting period, fish were lightly anaesthetized using benzocaine (50 mg l^−1^). Fish were immediately removed from anaesthetic solution once they had lost dorso-ventral equilibrium and did not respond to physical stimuli. Unique tags were delivered subcutaneously distally to the dorsal fin or on the operculum, depending on fish size. Fish were then transferred to a fully oxygenated recovery tank and observed until equilibrium was re-established before being returned to housing tanks. All individuals were given a minimum of two weeks to recover from handling stress before commencing testing. Tags remained visible throughout the entire experimentation period.

### Acclimation experimental design

Individual fish were randomly allocated to one of two acclimation temperature treatment groups: 20 ± 1°C and 28± 1°C. Holding tank temperatures were changed up or down to experimental temperatures slowly over 48 h to minimize thermal shock. Due to aggression at the high temperature, sub-adults and adults allocated to the 28°C treatment were housed in nine, 400 L recirculating tanks at lower densities. Fish were held at their respective temperatures for a minimum of four weeks prior to experimentation. Due to the small number of large fish available, some of the juveniles, the subadults and adults were acclimated to, and tested at, both temperatures. Fish were acclimated to one temperature for four weeks, tested and then acclimated to the opposite temperature for a further four weeks before final testing. The order of acclimation temperatures was randomized for individuals. Where individuals were used for multiple tests, they were given a minimum of two weeks recovery between tests.

### Maximum thermal tolerance (CT_max_)

*CT*_max_ was estimated by gradually exposing *M. peelii* to increasing water temperatures until reaching a critical threshold (defined as the loss of dorsoventral equilibrium). *M. peelii* were placed into a 100 L water bath containing oxygen-saturated water at their acclimation temperature and given 1 h to habituate to the conditions. Temperatures were regulated using a chiller (Hailea HC-1000A, Guangdong Hailea Group Co., Ltd, China) and two 600 W submersible heaters (Schego, Schemel & Goetz GmbH & Co., Germany). Oxygen saturation levels (% air saturation) were maintained above 90% throughout *CT*_max_ determinations. Oxygen concentrations and water temperatures were monitored throughout trials using an oxygen dipping probe and temperature probe connected to a Fibox 3 (DP-PSt3, PreSens, Regensburg, Germany). A customized permeable cover was placed 1 cm below the surface of the water to prevent fish from performing ASR during the trials. A submersible pump (600 L h^−1^ Moray 360, Aqua One Pty Ltd, Australia) ensured water within the water bath was well mixed. Water temperature was increased at a rate of 0.2°C min^−1^ until fish were observed to lose equilibrium and were unable to maintain dorso-ventral orientation at which point *CT*_max_ was recorded. This heating rate was chosen as it was slow enough to allow comparisons of *CT*_max_ across individuals that varied substantially in size and therefore have different thermal masses (Becker and Genoway, 1979). Fish were immediately transferred to an aerated recovery tank at their tank acclimation temperature until equilibrium was re-established. Fish ID, standard length (mm), total length (mm) and body mass (g) of each fish was recorded after recovery and prior to being returned to their respective holding tank.

### Oxygen limited thermal tolerance (PCT_max_)

To determine how oxygen availability influences the maximum thermal tolerance of *M. peelii,* P*CT_max_* was estimated using a modified *CT*_max_ protocol (Ern *et al.,* 2016). In addition to *CT*_max_ measures made at 100% air saturation, *CT*_max_ was also measured at 50, 30 and 16% air saturation consistent with the range of aquatic oxygen saturation levels measured in *M. peelii* habitats in the Queensland Murray Darling Basin (Fig. 2). Experimental oxygen saturation levels were obtained by bubbling nitrogen gas into the water bath to displace oxygen and water temperatures were maintained at the acclimation temperature of the fish using a chiller or aquarium heaters. Once the experimental oxygen saturation level was reached, fish were transferred from their holding tanks into the water bath and given 1 h to habituate to the experimental environment. A standard *CT*_max_ trial was then conducted as described above, and upon loss of equilibrium fish were transferred to a recovery tank prior to being returned to their holding tanks.

### Hypoxia loss of equilibrium threshold (O_2,LOE_), ASR and GVR

Hypoxia tolerance was estimated by gradually reducing aquatic oxygen saturation levels in a test tank holding fish (*n* = 16–25 per temperature treatment, mass = 0.55–2500 g). Fish were transferred into the test tank (40 x 40 x 60 cm), maintained at normoxia (100% air saturation) using an air stone and oxygen cylinder. Fish were given 1 h to habituate to the test tank. Temperature was regulated using a chiller and/or submersible heaters. Temperature and oxygen saturation levels were monitored throughout the trials as detailed above. A small water pump (Aqua One, Sydney, NSW, Australia) was placed inside the testing tank to ensure water was well mixed. A Perspex cover was placed on the surface of the water to prevent mixing of atmospheric oxygen with the tank water and to prevent *M. peelii* from performing ASR.

Observations were made using four fish simultaneously, for animals <150 g. Larger *M. peelii* were tested individually. All observations were made through one side of the tank and covered with mirror film that prevented fish from being disturbed by the observer; the remaining tank was covered with black fabric. At the onset of testing, nitrogen gas was bubbled through the tank to remove oxygen from the water. The rate of oxygen decline was regulated throughout the trial to maintain a steady decline in oxygen from 100% air saturation until fish lost equilibrium over approximately 2 h following McNeil and Closs (2007). GVRs, as the frequency of opercular movements, were recorded for 15 s every 10 min until ASR was attempted, after which sampling occurred every 5 min until O_2,LOE_ was reached. The oxygen saturation level at which *M. peelii* were observed to first attempt ASR (ASR_crit_) was recorded as fish actively seeking to access to the top 2 cm of the water surface. Once fish were observed to lose dorso-ventral orientation, oxygen saturation level was recorded as O_2,LOE_ and fish were rapidly removed from the tank and placed into an aerated recovery tank. Once fish had recovered, fish ID, standard length (mm), total length (mm) and body mass (g) were recorded before being returned to holding tanks.

### Blood oxygen transport capacity metrics and blood lactate responses to acute hypoxia

To assess whether thermal acclimation increased the oxygen transport capacity of sub-adult fish and reduced their reliance on anaerobic metabolism during hypoxia, a subset of the fish used in above trials were acutely exposed to either normoxic conditions or hypoxia (20% air saturation) for 2 h (*n* = 7–8 per treatment; mass = 81–203 g) at their acclimation temperature. Exposures occurred in the holding tanks to minimize changes in blood variables associated with handling stress. Following 2 h of exposure, fish were netted from their tanks and then rapidly anaesthetized in benzocaine (500 mg l^−1^). A blood sample was collected from the caudal vein into a heparinized syringe. A 3 μl sample was analysed immediately using a hand-held lactate meter (Lactate Pro 2, Kyoto, Japan). An additional 5 μl sample was diluted immediately 1:10 in distilled water and stored on ice for subsequent haemoglobin determination using a commercially available kit (ab234046; Abcam, Melbourne, Vic. Australia). The remaining sample was transferred into microhematocrit tubes, sealed and centrifuged at 14000 g for 3 min. Haematocrit was determined as the percentage of red blood cells in the sample.

### Statistical analyses

All data were analysed using the statistical software R version 3.6.0 (R Core Team, 2019). *CT*_max_ and P*CT_max_* data required the use of a generalized additive model (GAM) using the ‘mgcv’ package (Wood *et al.,* 2016). The combined effects of aquatic oxygen saturation level and body mass (g) resulted in different regression patterns that could not be accurately modelled using either linear or polynomial mixed effects models (Table 1). GAM was confirmed as best model fit following model comparisons between a GAM, a linear mixed effects model and a linear mixed effects model with a quadratic term using an Akaike Information Criterion (AIC) and F-test from an ANOVA. Plots of residual vs fitted values and histogram of residuals showed approximate normal distribution following log-transformation of *Body Mass* in the model. *Hypoxia treatment* and *Acclimation temperature* were treated as fixed factors in the model combined with a non-parametric smoother fitted to log *(Mass)*. Individual *Fish ID* was incorporated into the model as a random effect using a smoothing function. Post-hoc analyses were conducted using estimated marginal means from the ‘emmeans’ package (Lenth, 2019) to investigate the overall effects of hypoxia treatment on *CT*_max_ within and between acclimation temperatures.

**Table 1:** Summary statistics from linear and quadratic models of the effects of body mass and acclimation temperature on upper thermal limits (*CT*_max_) in *M. peelii* at each level of oxygen concentration

		Mean Sq	Num DF	Den DF	*F*	*P*
100% air saturation						
Best fit model:	Linear					
	Acclimation temperature	3.71	1	52.99	1304.2	2.20e^−16^
	log mass	0.01	1	53.01	26.98	3.34e^−06^
50% air saturation						
Best fit model:	Linear					
	Acclimation temperature	165.68	1	53	1820.3	2.20e^−16^
	log mass	3.901	1	53	42.86	2.42e^−08^
30% air saturation						
Best fit model:	Polynomial					
	Acclimation temperature	39.81	1	6.85	234.65	1.49e^−06^
	log mass	3.99	1	12.22	23.49	<0.001
	poly(log Mass)	1.91	1	13.33	11.23	0.005
16% air saturation						
Best fit model:	Polynomial					
	Acclimation temperature	101.87	1	48.74	228.93	2.20e^−16^
	log mass	100.56	1	49.66	225.98	2.20e^−16^
	poly(log Mass)	32.34	1	50.05	72.674	2.57e^−11^

To further examine the nature of the relationship between *CT*_max_ and body mass at each level of oxygen saturation, separate linear mixed effects models with and without a second-order polynomial function (quadratic) were fitted using the ‘nlme’ package (Pinheiro *et al.,* 2019). *Acclimation* temperature was treated as a fixed factor in the models, with *Fish ID* treated as a random effect. AIC scores were compared between models to determine best fit.

A linear mixed effects model was used to analyseO_2,LOE_ ASR_crit_ and GVR data using the ‘lmerTest’ package (Kuznetsova *et al.,* 2017). *Acclimation* temperature and *Treatment* temperature were treated as fixed factors in the model, log body mass was a continuous covariate and *Fish ID* and *experimental trial number* were treated as random factors. *Oxygen saturation level* was used as an additional covariate in the GVR model. A third-order polynomial transformation of *oxygen saturation level* was applied following model comparisons using AIC. Post-hoc analyses comparing pairwise differences in O_2,LOE*,*_ ASR and GVR between acclimation and treatment groups were conducted using estimated marginal means within the ‘emmeans*’* package (Lenth, 2019).

**Figure 3:**
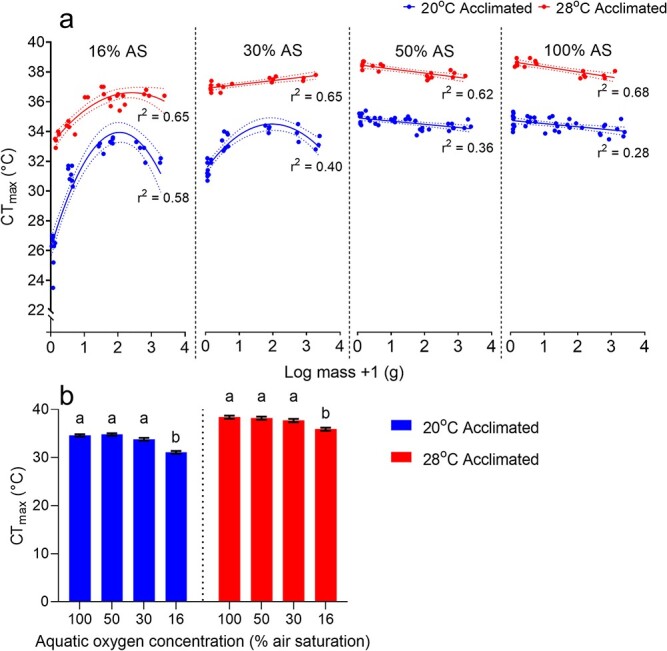
(a) The relationship between body mass (g) and oxygen-dependent thermal tolerance in (P*CT*_max_) in *M. peelii* acclimated to 20°C (blue) and 28°C (red). There was a weak negative relationship between body mass and *CT*_max_ at normoxia and 50% air saturation (AS); however, the relationship became strongly nonlinear at 30% and 16% air saturation, with smaller fish having a lower oxygen dependent maximum thermal tolerance than larger fish. Data points represent values from individual fish. Lines represent best fit models (linear and second-order polynomial) and 95% confidence intervals. (b) Estimated marginal means for the effects of aquatic oxygen level on upper thermal limits in *M. peelii.* Treatments with the same letter are not significantly different.

Blood oxygen transport metrics (HCT and Hb) and blood lactate levels were analysed using a multivariate linear model with *oxygen saturation level* and *acclimation temperature* as fixed factors, *body mass* as a continuous covariate. All blood parameters (haematocrit, haemoglobin, lactate) were analysed simultaneously using the *cbind* function. Main effects of body mass, temperature and oxygen saturation level were determined using the anova function from the *car* package (Fox and Weisberg, 2019).

All figures were produced using GraphPad Prism Version 8.4.1.

## Results

### Waterhole water quality assessments

The water depth varied substantially depending on season, with Weribone and Elgin waterholes being deeper in summer compared to Reilly’s Weir which was deeper in spring (Fig. 2). Water temperature was consistently higher across all waterholes in summer compared to spring, with temperatures being highest in the afternoon (range: Weribone: 19–32°C; Reillys weir: 23-36°C; Elgin waterhole: 24–32°C). Oxygen saturation was observed to have greater variation between morning and afternoon samples between seasons, with oxygen saturation being more variable during summer compared to spring (range Weribone: 8.5%–103% air saturation; Reillys weir: 23–210% air saturation; Elgin waterhole: 11%–115% air saturation). With the exception of Weribone during spring, the water column was highly stratified, with both temperature and oxygen saturation reducing with depth.

### Maximum thermal tolerance (CT_max_) and oxygen limited thermal tolerance (PCT_max_)

There was an overall significant interaction between acclimation temperature and oxygen concentration level on *CT*_max_ in *M. peelii* (GAM; *F* = 3.52, *P* = 0.019, *df* = 3). There were also significant main effects of both acclimation temperature (GAM; *F* = 242.31, *P* < 0.001, *df* = 1) and oxygen concentration (GAM; *F* = 73.02, *P* < 0.001, *df* = 3) on *CT*_max_. There was a significant effect of the smoothing terms log fish mass and fish ID (GAM; Log mass: *E df* = 4.72, *Ref df* = 5.15, *F* = 14.63, *P* < 0.001; Fish ID: *E df* = 127.69, *Ref df* = 182, *F* = 2.51, *P* < 0.001). At oxygen concentrations at or above 50% air saturation, there was a significant negative linear relationship between mass and *CT*_max_ for both acclimation groups (Fig. 3, Table 1). However, at oxygen tensions below 50% air saturation, the relationship between *CT*_max_ and body mass became increasingly non-linear. In the 20°C acclimation group, a significant non-linear relationship between body mass and *CT*_max_ was evident at both 30% and 16% air saturation while for the 28°C acclimated group the relationship remained linear at 30% air saturation but became strongly non-linear at 16% air saturation. In both acclimation groups, the nonlinear relationship between body mass and thermal tolerance resulted in both smaller and larger *M. peelii* having lower measures of *CT*_max_ relative to mid-sized fish.

Estimated marginal means were used to compare the overall effects of acclimation temperature and aquatic oxygen level on upper thermal limits in *M. peelii*. Acclimation to 28°C significantly increased *CT*_max_ compared to individuals acclimated to 20°C (Fig. 3). Within both temperature acclimation groups, the reduction in oxygen saturation level from normoxia to 50% air saturation did not significantly affect *CT*_max_. However, *CT*_max_ was significantly lower at both 30% and 16% air saturation with respect to normoxia.

### Hypoxia tolerance

**Figure 4:**
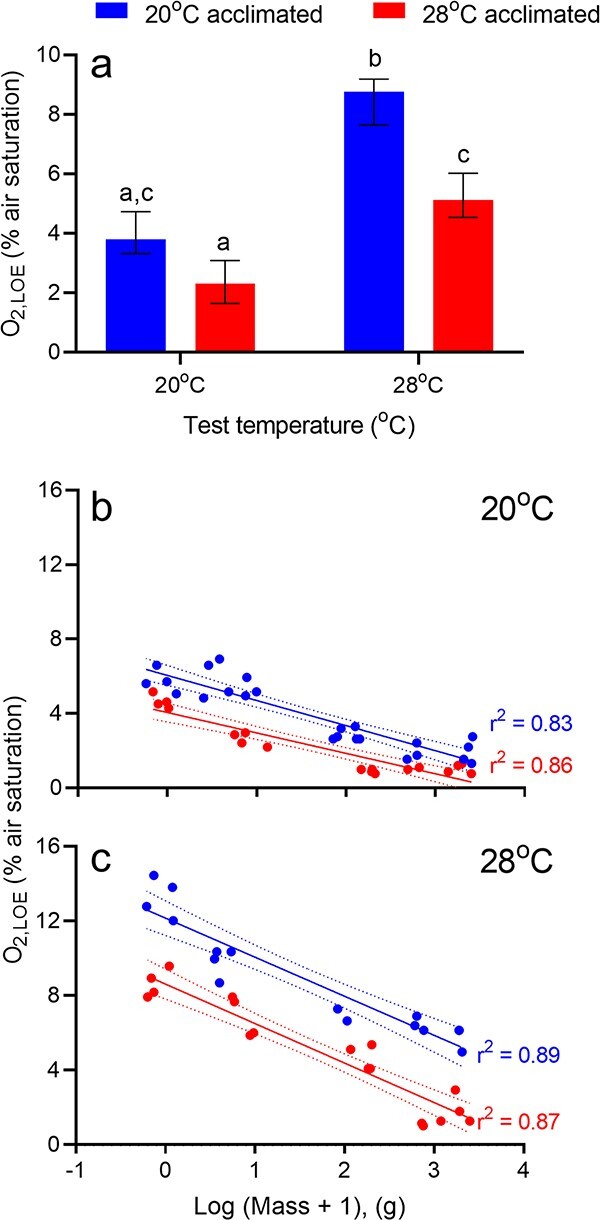
(a) The effect of acclimation temperature on O_2,LOE_ in *M. peelii*. Animals were tested at both their acclimation temperature (denoted by colours: blue = 20°C acclimated, red = 28°C acclimated) and acutely at the opposing temperature. Bars denote estimated marginal means ± upper and lower confidence intervals. Letters that differ represent treatments that are statistically significant from others (*P* < 0.05). (b,c) The relationship between body mass (g) and the aquatic oxygen level (% air saturation) at which *M. peelii* lost equilibrium (O_2,LOE_) in fish acclimated to either 20°C (blue) or 28°C (red) and tested at 20°C (b) or 28°C (c). *N* = 16–25 per temperature. Dotted lines represent 95% confidence intervals.

O_2,LOE_ was significantly affected by body mass, test temperature, acclimation temperature and the interaction between acclimation and test temperature (Table 2). O_2,LOE_ was not significantly different be tween acclimation groups when tested at their acclimation temperatures, however, O_2,LOE_ of fish acclimated to 20°C and tested acutely at 28°C doubled from 4.08 ± 0.34 to 8.41 ± 0.37% air saturation (Fig. 4a). Conversely, *M. peelii* acclimated to 28°C and tested at acutely 20°C had a lower O_2,LOE_ (2.37 ± 0.34% air saturation) than fish acclimated to and tested at 28°C (5.20 ± 0.35% air saturation). Measures of O_2,LOE_ were found to vary significantly depending on fish body mass (Fig. 4b, c). Independent of acclimation or test temperature, there was a significant negative relationship between body mass and O_2,LOE_*,* indicating that smaller individuals were less tolerant of acute hypoxia than larger fish. Fitting a power function to the entire raw data set revealed that O_2,LOE_ scaled to body mass with an exponent of ~ 0.19.

**Table 2:** Summary statistics from linear mixed effects models comparing the effects of acclimation temperature, test temperature and body mass (log transformed) on O_2,LOE_ in *M. peelii*

	F Statistic	DF (num, den)	*P*
Body mass (log)	123.02	1, 28.15	<0.001
Test temperature	85.94	1, 28.12	<0.001
Acclimation temperature	45.62	1, 28.12	<0.001
Test x acclimation temperature	10.39	1, 28.11	0.003

**Table 3:** Summary statistics of linear mixed effects models examining the relationships between acclimation temperature, test temperature and body mass on air-surface respiration threshold (ASR) in *M. peelii*

	F Statistic	*DF (num, den)*	*P*
Body mass (log)	3.76	1, 24.95	0.064
Test temperature	23.19	1, 25.06	<0.001
Acclimation temperature	10.89	1, 25.06	0.003
Test x acclimation temperature	6.22	1, 25.05	0.02

**Figure 5:**
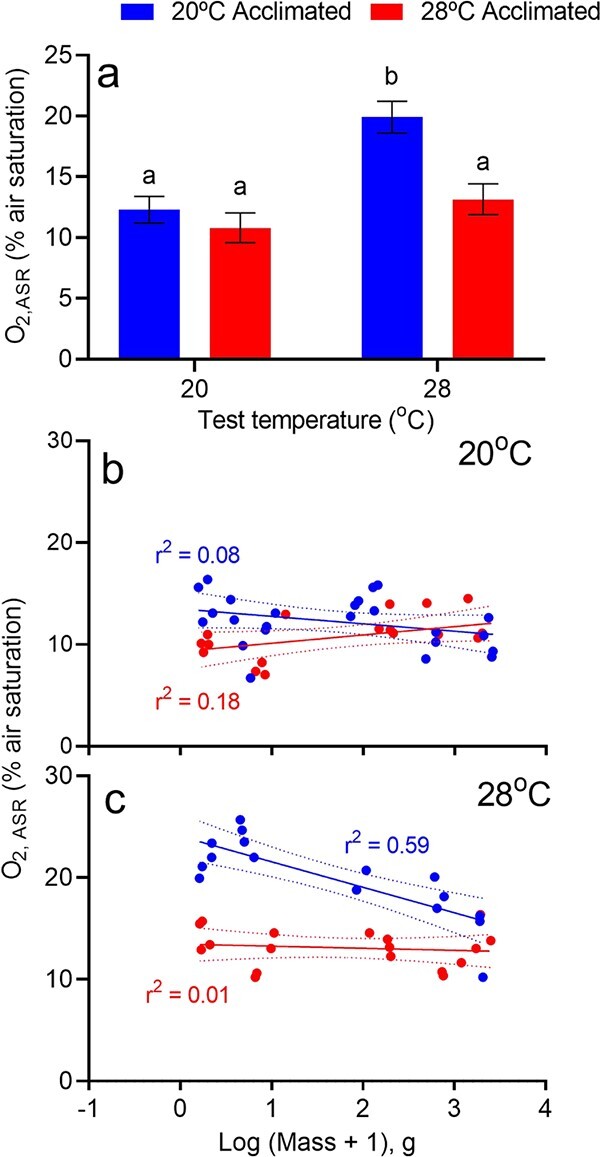
(a) The effect of temperature on the aquatic oxygen level (% air saturation) at which *M. peelii* first utilized aquatic surface respiration (ASR threshold). Bars denote estimated marginal means ± upper and lower confidence intervals. Treatments with the same lowercase letters are not signficantly different from each other (P > 0.05). (b,c) The relationship between body mass (g) and ASR threshold in *M. peelii* acclimated to 20°C (blue) or 28°C (red) for 4 weeks. Fish were exposed to a gradual decline in aquatic oxygen level, and tested at both 20°C (a) and 28°C (b) (*n* = 16–25 per temeprature). Dotted lines represent 95% confidence intervals.

There were significant main effects of test temperature and acclimation temperature, and an interaction between the two, on the oxygen tension at which fish first attempted ASR (Table 3; Fig. 5). The effect of body mass on these relationships was not significant. The interaction between test temperature and acclimation temperature was driven largely by the behaviour of 20°C-acclimated *M. peelii* with fish tested acutely at 28°C attempting ASR at higher oxygen saturation levels than 28°C-acclimated fish tested at 28°C. ASR threshold was not significantly different between 20°C and 28°C acclimated fish tested at their acclimation temperatures nor between 28°C-acclimated fish tested at either 20°C or 28°C.

**Figure 6:**
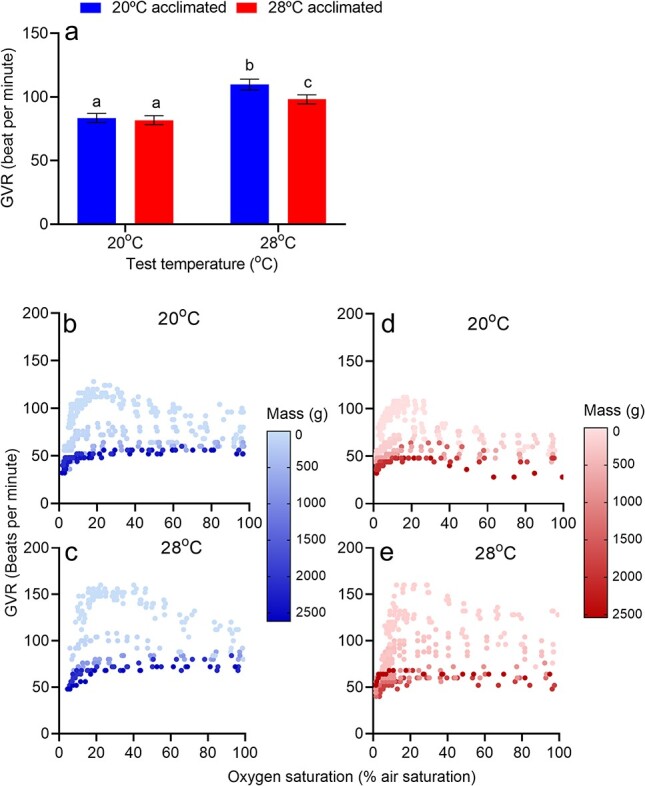
(a) Estimated marginal means for the effects of aquatic oxygen level, acclimation and test temperatures on GVR in *M. peelii.* Fish were acclimated to either 20°C (blue) or 28°C (red) for 4 weeks and then exposed to declining aquatic oxygen saturation levels at both 20°C and 28°C. Bars denote estimated marginal means ± upper and lower confidence intervals. Treatments with the same lowercase letters are not significantly different from each other (P > 0.05). (b–e) Body-size dependent GVR in *M. peelii* (body size range = < 1 g—> 2500 g) acclimated to 20°C (blue) and 28°C (red) and tested at both 20°C (b, c) and 28°C (d, e).

There was a significant interactive effect of acclimation temperature and test temperature on GVRs (Table 4; Fig. 6). In both acclimation groups, GVR was highest when animals were tested at 28°C. No significant difference in GVR was detected between acclimation groups when fish were tested at 20°C. GVR was highly dependent on body mass. Smaller fish consistently maintained higher measures of GVR than larger fish independent of both acclimation and test temperature. However, this negative relationship between GVR and body mass was most apparent in both acclimation groups when tested at 28°C. At both test temperatures, smaller fish increased GVR as aquatic oxygen saturation levels declined until just before O_2,LOE_ when GVR declined precipitously. Conversely larger fish maintained GVR independent of aquatic O_2_ concentration until shortly before fish reached their O_2,LOE_point (~ 10% air saturation).

### Blood oxygen transport capacity metrics

**Figure 7:**
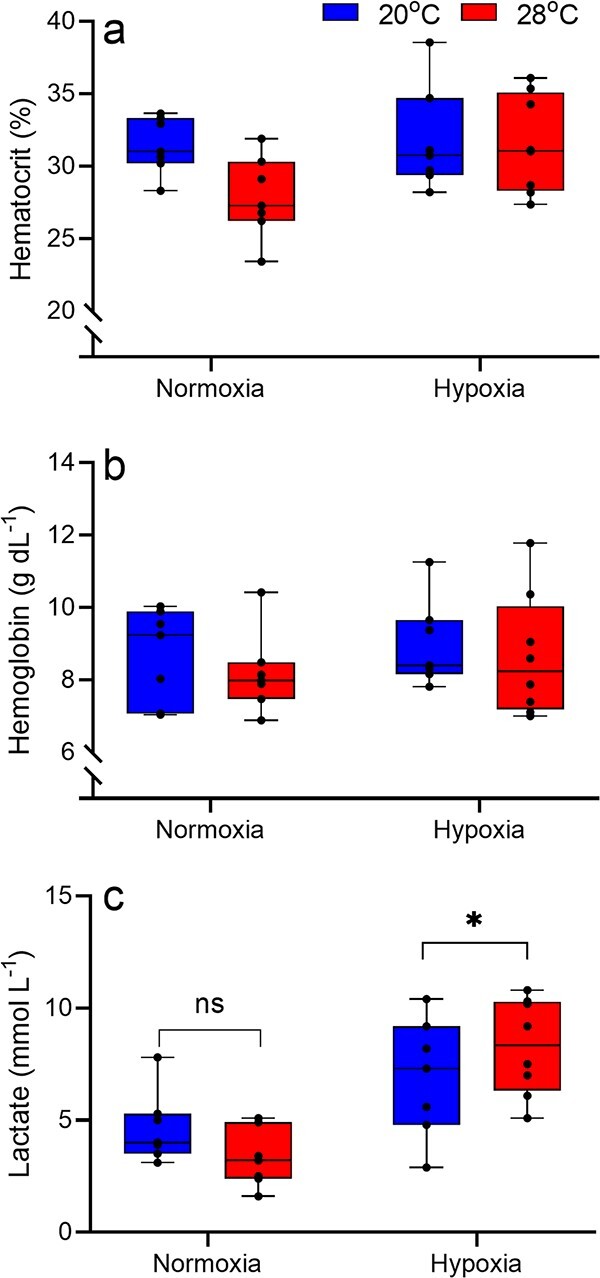
The effects of acclimation to either 20°C (blue) or 28°C (red) followed by an acute hypoxia exposure on blood oxygen transport metrics (haematocrit (a) and haemoglobin concentration (b)), and blood lactate levels (c) in juvenile *M. peelii*. Asterisks indicate treatment groups are statistically different from one another (*P* < 0.05).

Multivariate analyses showed that there was a significant main effect of oxygen concentration and a weakly significant interaction between acclimation temperature and oxygen concentration on blood metrics (Table 5). There was no main effect of body mass or acclimation temperature. The overall model effects were driven largely by blood lactate responses. Neither haematocrit nor haemoglobin concentration is affected by temperature acclimation or acute hypoxia exposure; there was also no interaction between the two factors (Table 6; Fig. 7a, b). Blood lactate concentration was however, significantly affected by hypoxia exposure and there was a significant interaction with acclimation temperature (Table 6). Blood lactate levels were significantly higher in animals exposed to hypoxia at 28°C relative to animals in normoxia at 28°C (Fig. 7c).

**Table 4:** Summary statistics of linear mixed effects models examining the relationships between acclimation temperature, test temperature, aquatic oxygen level (third-order quadratic function) and log body mass on gill ventilation rates in *M. peelii*

	F Statistic	DF (num, den)	*P*
Body mass (log)	643.22	1, 1254	< 0.001
Test temperature	176.47	1, 1254	< 0.001
Acclimation temperature	28.47	1, 72	< 0.001
Oxygen (poly, 3)	221.14	3, 1254	< 0.001
Test x acclimation temperature	8.66	1, 1254	0.003

## Discussion

Independent of body size, acclimation to increased water temperatures improved most metrics of both thermal and hypoxia tolerance in *M. peelii*. These responses correlated with a significant change in a suite of behavioural and physiological measures, including GVR and ASR which were both reduced. Absolute hypoxia tolerance limits (O_2,LOE_) were highly body-size specific in *M. peelii* with larger fish being more hypoxia tolerant than smaller fish; however, the relationship between body size and oxygen-dependent thermal tolerance was far more complex. Contrary to predictions, under normoxic conditions smaller *M. peelii* were only marginally more thermally tolerant than larger individuals, however, under conditions of low oxygen availability, the relationship between body size and thermal tolerance suggested that both the largest and the smallest fish were disproportionately affected. Taken together, this study shows that while thermal acclimation can improve acute thermal and hypoxia tolerances in *M. peelii*, the significant allometric scaling relationship for oxygen-dependent thermal tolerance means that both the smallest and the largest life stages are likely to be at higher risk from combined high temperature and low aquatic oxygen stress.

Consistent with our hypotheses, hypoxia and upper thermal tolerances were highly sensitive to contemporary and antecedent environmental temperatures in *M. peelii*. Hypoxia tolerance was acutely thermally sensitive, declining with increasing temperature, and showed a significant degree of thermal plasticity (acclimation capacity). Likewise, protracted exposure to elevated temperatures increased upper thermal limits. Consistent with other studies (e.g. Hughes and Saunders, 1970; Giacomin *et al.,* 2019), we observed that fish significantly increased their GVR and the amplitude of opercular movements with increasing water temperature to increase oxygen supply to metabolizing tissues to delay the onset of anaerobic metabolism. Moreover, acute exposure of fish to warm water meant that fish engaged ASR earlier at higher oxygen saturation levels. Although these mechanisms do assist in meeting increased oxygen requirements at high temperature, they are also likely to incur both energy and fitness costs with protracted exposure to these environmental conditions. Thermal acclimation of physiological function can reduce costs associated with prolonged exposure to ‘sub-optimal’ temperatures (Wilson and Franklin, 2002). As observed, acclimation to elevated water temperatures improved metrics of hypoxia tolerance and upper thermal tolerance limits across all *M. peelii* size classes. Fish species that inhabit environments that experience a broad range of thermal conditions are more likely to display thermal plasticity than those that do not; however, the frequency and magnitude of thermal fluctuations determine the nature of the plasticity responses (e.g. Niehaus *et al.,* 2006, 2011; Beaman *et al.,* 2016; Shah *et al.,* 2017). The significant changes in aquatic temperature and oxygen saturation levels measured both diurnally and seasonally in *M. peelii* habitat (Fig. 2) would suggest that *M. peelii* routinely experience the necessary environmental pressures that would drive the acclimation of performance with changing environmental conditions. Additionally, acclimation to high temperature water resulted in improved hypoxia tolerance relative to fish acclimated to cooler waters, indicating a cross-tolerance relationship between these stressors in this species. Cross-tolerance occurs when physiological responses to one stressor improve their tolerance to a subsequent stressor (Todgham and Stillman, 2013). The mechanistic basis for thermal acclimation-induced improvements in hypoxia tolerance can include increases in gill surface area (Sollid and Nilsson, 2006; Anttila *et al.,* 2015; McBryan *et al.,* 2016; Gibbons *et al.,* 2018), cardiac remodelling (Egginton, 2002, Klaiman *et al.,* 2011, Anttila *et al.,* 2015), increased aerobic (red) muscle volume (Rome *et al.,* 1984; Young and Egginton, 2009) and increases in oxygen carrying capacity (e.g. Neale *et al.,* 1977; Wells *et al.,* 1989; Wells, 2009; Jayasundara and Somero, 2013). In *M. peelii*, changes in haematocrit and haemoglobin concentration did not appear to underpin the improvement in thermal tolerance; future studies should examine changes to gill surface area metrics and haemoglobin oxygen binding affinities in *M. peelii*, which have been shown to improve oxygen supply and delivery (offloading) at tissues in other fish species (Cook *et al.,* 2013; Lilly *et al.,* 2015).

Body size contributes significantly to variations in hypoxia and thermal tolerance in many fish species, although the nature of the relationships seems to be highly species-specific. Generally, larger fish are thought to be more hypoxia tolerant than smaller fish, but more thermally sensitive (Nilsson and Ostlund-Nilsson, 2008; Roze *et al.,* 2013). The capacity to ‘tolerate’ low oxygen environments likely reflects the complexity of physiological and behavioural traits/strategies underpinning measures of tolerance in fish. For example, in *A. ocellatus*, developmental changes in their underlying metabolic physiology (energy metabolism), specifically an increase in their absolute anaerobic potential, correlates positively with growth meaning that larger fish have a greater absolute capacity to withstand hypoxic water than smaller (younger) fish because they can better meet more of their energy requirements anaerobically (Almeida-Val *et al.,* 2000). Alternatively, where smaller fish have a lower absolute anaerobic capacity than larger fish, a lower P_Crit_ means that smaller fish can delay the point at which energy demands need to be met via anaerobic metabolism (Almeida-Val *et al.,* 2000; Nilsson and Ostlund-Nilsson, 2008; Urbina and Glover, 2013). Consistent with the aforementioned pattern, small *M. peelii* displayed lower hypoxia tolerances at both acclimation temperatures. Increases in hypoxia tolerance with increasing fish size likely reflect mass-specific increases in both anaerobic capacity and absolute oxygen uptake capacity (gill surface area) as seen in several other fish species (e.g. Nilsson and Ostlund-Nilsson, 2008; Urbina and Glover, 2013; Scheuffele *et al.,* 2021). Increasing GVRs and early engagement of ASR behaviour indicated that smaller *M. peelii* utilized additional hypoxia avoidance strategies to avoid having to resort to anaerobic metabolism. However, this extra activity likely compounded their metabolic demands particularly at high temperature, placing further pressure on aerobic supply capacity. Whether this also means that smaller *M. peelii* may be able to remain aerobic for slightly longer than larger fish (i.e. lower P_Crit_), as seen in marine red drum, *Sciaenops ocellatus* (Pan *et al.,* 2016) is unclear from this study, but ultimately does not affect lethal tolerance limits.

**Table 5:** Type II MANOVA Pillai test statistics of the effects of acclimation to either 20 °C or 28°C (‘acclimation temperature’), oxygen saturation level (hypoxia or normoxia) and their interaction on blood oxygen transport capacity metrics (hemoglobin and hematocrit) and blood lactate levels. Body mass was considered as continuous covariate in the model

	Test Statistic	~F (nDF, dDF)	*P*
Mass	0.20	1.85 (3, 22)	0.17
Acclimation temperature	0.13	1.13 (3, 22)	0.35
Oxygen saturation level	0.53	8.41 (3, 22)	<0.001
Temperature ^*^ oxygen	0.18	1.57 (3, 22)	0.23

**Table 6:** Multivariate ANOVA test statistics examining the effects of acclimation to either 20 °C or 28°C (‘temperature’), oxygen saturation level (hypoxia or normoxia) and their interaction on blood oxygen transport metrics (hemoglobin and hematocrit) and blood lactate levels

	Estimate	SE	*t*	*P*
Hematocrit				
Mass	0.003	0.018	0.186	0.854
Temperature	−0.212	1.617	−0.131	0.897
Oxygen saturation level	−0.334	1.651	−0.202	0.841
Temperature ^*^ oxygen	−3.396	2.332	−1.456	0.158
				
Hemoglobin				
Mass	−0.014	0.008	−1.836	0.079
Temperature	−0.535	0.68	−0.788	0.439
Oxygen saturation level	−0.288	0.694	−0.415	0.682
Temperature ^*^ oxygen	0.144	0.981	0.147	0.885
				
Lactate				
Mass	0.018	0.012	1.560	0.132
Temperature	1.598	1.013	1.578	0.128
Oxygen saturation level	−2.272	1.034	−2.198	0.038
Temperature ^*^ oxygen	−3.106	1.461	−2.127	0.044

In contrast to hypoxia tolerance, increasing body size limits upper thermal tolerances in many aquatic species (e.g. Recsetar *et al.,* 2012; Roze *et al.,* 2013; Roman *et al.,* 2019). Our data support a role for body size-dependent changes in thermal tolerance in *M. peelii* but indicate that the nature of the relationship between body size and thermal tolerance is highly dependent on ambient oxygen concentration. Under normoxic and mildly hypoxic conditions, there was a significant, albeit small (<0.2), negative scaling relationship between body mass and *CT*_max_ in *M. peelii* with smaller fish having higher *CT*_max_ than larger fish. Nonetheless, whether this scaling relationship is maintained for larger *M. peelii*, given that animals can grow up to 100 kg, is unknown. In contrast, under moderate to severe hypoxia, the relationship reversed direction and became increasingly non-linear, suggesting that there was a body-size-dependent synergistic relationship between severe hypoxia and temperature in this species. Under severe hypoxia, low anaerobic capacity and age-specific performance constraints may constrain the thermal tolerance of small *M. peelii* while oxygen uptake and/or cardiovascular supply limitations, or competing tissue demands for energy (e.g. by reproductive tissues) may constrain upper thermal limits in larger fish (Pörtner and Farrell, 2008; Peck *et al.,* 2009; Ohlberger, 2013). The magnitude of the effect of hypoxia on thermal tolerance was greatest in 20°C-acclimated fish, since chronic exposure to warmer temperatures likely resulted in compensatory changes to oxygen uptake and/or cardiovascular capacities that improved the performance of warm-acclimated fish. Our study indicated that, accounting for the influence of body size, P*CT*_max_ was between 30 and 16% air saturation for *M. peelii*. This value is broadly consistent with values recorded for several other fish species (e.g. red drum: *Sciaenops ocellatus,* ~27% air saturation; Ern *et al.,* 2016; and black-axil chromis: *Chromis atripectoralis*, ~29% air saturation; five-lined cardinalfish: *Cheilodipterus quinquelineatus*, ~25% air saturation; and spiny chromis damselfish: *Acanthochromis polyacanthus*, 33% air saturation; Ern *et al.,* 2017), but examining the thermal tolerance of *M. peelii* at more oxygen saturation levels between 3% and 16% could better define the specific *PCT_max_* point for the species. A *PCT_max_* of between 30% and 16% air saturation suggests that in normoxic and moderately hypoxic waters, the thermal tolerance of *M. peelii* is likely to be largely oxygen-independent. However, in severely hypoxic waters thermal tolerance is substantially constrained, and the degree of constraint is greatest for the smallest and to a lesser extent, the largest fish. This has significant ramifications for the management of fish in waterholes that become highly stratified and oxygen depauperate during protracted low flow and no flow periods.

Our brief survey of three waterholes in the Queensland Murray-Darling Basin in spring and summer of 2019–2020 demonstrated the capacity of these refugia to become physiologically challenging for resident *M. peelii*. Although the maximum recorded water temperatures remained below the upper lethal limits (*CT*_max_) of *M. peelii*, the thermal safety margin in summer was just 2°C–6°C and 5°C–10°C in spring, indicating that this species is living close to its thermal limits. Similarly, the deeper waterholes became highly stratified in late summer and oxygen saturation levels dropped to as low as 8% air saturation which is approaching the lethal limit (O_2,LOE_) for both small and large *M. peelii*. At these very low oxygen saturation levels, the thermal tolerance of fish is also likely to be suppressed. The proximity of fish to their physiological limits increases the risk that an acute destratification event (e.g. following sudden rainfall), algal bloom or acute heat wave will precipitate a large fish death event. Our data suggest that in any of these scenarios, small fish are likely to be disproportionately affected, but larger fish are also vulnerable. Mass fish death events attract considerable public outcry particularly when large mature fish are involved (e.g. the Menindee Lakes fish kill of 2018–2019; Moritz *et al.,* 2019); yet our data suggest that loss of smaller fish may be a greater likelihood, but could go relatively unnoticed due to their small size. While the loss of the larger breeding fish from a population is undoubtedly significant for population persistence, the loss of large numbers of juveniles can be equally catastrophic (Downes *et al.,* 2021) particularly in slow growing, long-lived species like *M. peelii*, since their loss may go unrecognized for many years (Keevil *et al.,* 2018). The present study examined the thermal tolerances of fish up to 2.5 kg, but adult *M. peelii* can grow up to 100 kg. Future studies are needed to determine the specific thermal tolerance limits of the largest individuals in the population.

Comparing thermal tolerance limits across a substantial body size range required *M. peelii* to be tested at a rapid, but consistent heating rate (0.2°C min^−1^); however, such rapid heating rates may overestimate the upper thermal limits of large fish if there is a lag between water temperature and the core body temperature of the fish (Becker and Genoway, 1979). It is possible then that the observed relationships between thermal tolerance and body mass for *M. peelii* may be an underestimate if the heating rate of the largest fishes did not keep pace with changes in core body temperature. In addition, ecological validity of data collected via conventional rapid heating *CT*_max_ protocols has been questioned since these protocols avoid the accumulation of thermal damage that would occur if fish were exposed to slower, more ecologically realistic heating rates (e.g. Rezende and Bozinovic, 2019, Blasco *et al.,* 2020, Lefevre *et al.,* 2021, Bartlett *et al.,* 2022; Ørsted *et al.,* 2022). Thermal tolerance protocols that employ slower heating rates, such as the incremental thermal maximum (heating rates of ~0.2°C per day), report thermal limits that are several degrees lower than that recorded using conventional *CT*_max_ tests (e.g. Bartlett *et al.,* 2022). Likewise, studies that use the point at which physical performance becomes compromised (e.g. swimming capacity) as the endpoint instead of LOE also report lower thermal maxima than in standard *CT*_max_ protocols (e.g. Blasco *et al.,* 2020). Consequently, the upper thermal tolerance limits of *M. peelii* reported here may reflect an overestimate of the actual thermal tolerance maxima of the species. This has implications for predicting the capacity of the species to tolerate both sustained environmental warming and acute heatwave events. More studies are required to determine how heating rates and performance endpoints affect thermal tolerance limits, across the substantial body size range of this species and in conjunction with varying environmental oxygen levels.

Whilst our study demonstrated a capacity for *M. peelii* to thermally acclimate to ecologically relevant water temperatures in the laboratory, whether *M. peelii* actually thermally acclimate to the same extent in a natural setting is unknown. Further, it is unknown whether ontogenetic differences in habitat use may result in different oxygen and thermal experiences and therefore differences in acclimation responses between juvenile and adult fish. Adult *M. peelii* have been found to preferentially select the lower 15% of the water column (Koehn, 2009a; Koehn, 2009b) where both water temperatures and aquatic oxygen saturation levels are lowest. In contrast, hypoxia intolerance may mean that smaller fish avoid areas where aquatic oxygen saturation levels are low which may eliminate depth-related thermal refuges and increase their exposure to higher water temperatures (some of the waterholes surveyed in our study actually became hyperoxic in summer, but these were very shallow and consequently were very warm). Small fish may seek to surface breathe amidst emergent or floating vegetation to reduce the predation risk, or, under flowing conditions, may seek out areas where oxygen is being introduced to the water column (e.g. turbulence or respiring vegetation). However, in the context of a drying waterhole, these options maybe unavailable. Waterholes inhabited by *M. peelii* may also be hypoxic over a large proportion of their volume meaning that smaller animals likely contend with mild to moderate hypoxia for extended periods. Prolonged exposure to even mild hypoxia can induce compensatory responses that improve hypoxia tolerances in *M. peelii* (Gilmore *et al.,* 2019), which may allow animals to utilize areas of the waterhole that would appear suboptimal on the basis of the results of our study. Further work is needed to understand how acclimation to low aquatic oxygen saturation levels alters thermal tolerances and influences habitat usage in *M. peelii.*

## Conclusions

Collectively, the data presented in this study demonstrate that prior acclimation to elevated water temperatures improves the thermal and hypoxia tolerance of *M. peelii.* More importantly, this study has shown that the tolerance of juvenile *M. peelii* to the combined pressure of elevated water temperatures and hypoxia is significantly lower than for larger individuals. A greater understanding of the specific physiological traits that are responsible for differences in tolerance with body mass would provide critical insight as to how populations may be affected in the environment. Future research should consider habitat utilization as a means to ameliorate or mitigate physiological stress in *M. peelii* especially during the summer months when waterhole conditions are at their most extreme. As average temperatures are generally higher at lower latitudes, juvenile *M. peelii* in the Queensland Murray Darling Basin may be particularly vulnerable to the effects of future climate change. Identifying waterholes that are at reduced risk of experiencing elevated water temperatures and hypoxic events needs to be prioritized to ensure this species can persist into the future.

## Data Availability

All data are freely available from the University of Queensland’s digital repository upon request. DOI https://doi.org/10.48610/356ca3f.
